# Exercise-Induced Skeletal Muscle Adaptations Alter the Activity of Adipose Progenitor Cells

**DOI:** 10.1371/journal.pone.0152129

**Published:** 2016-03-25

**Authors:** Daniel Zeve, Douglas P. Millay, Jin Seo, Jonathan M. Graff

**Affiliations:** 1 Department of Developmental Biology, University of Texas Southwestern Medical Center, Dallas, Texas, United States of America; 2 Department of Molecular Biology and Center for Regenerative Science and Medicine, University of Texas Southwestern Medical Center, Dallas, Texas, United States of America; 3 Department of Medicine, Division of Endocrinology, University of Texas Southwestern Medical Center, Dallas, Texas, United States of America; Tohoku University, JAPAN

## Abstract

Exercise decreases adiposity and improves metabolic health; however, the physiological and molecular underpinnings of these phenomena remain unknown. Here, we investigate the effect of endurance training on adipose progenitor lineage commitment. Using mice with genetically labeled adipose progenitors, we show that these cells react to exercise by decreasing their proliferation and differentiation potential. Analyses of mouse models that mimic the skeletal muscle adaptation to exercise indicate that muscle, in a non-autonomous manner, regulates adipose progenitor homeostasis, highlighting a role for muscle-derived secreted factors. These findings support a humoral link between skeletal muscle and adipose progenitors and indicate that manipulation of adipose stem cell function may help address obesity and diabetes.

## Introduction

Obesity, diabetes, and associated metabolic sequelae are widespread and thus, additional therapeutic strategies are needed. A variety of studies indicate that a consistent exercise regimen, combined with a healthy diet, is an effective therapy [1–4]. Unfortunately, numerous factors limit the utility of this strategy. For instance, exercise is often physiologically and psychologically strenuous, and hectic modern life is not conducive to the long-term commitment needed to improve obesity and diabetes and their clinical outcomes [5]. Therefore, elucidation of the molecular mechanisms that underlie the benefits of exercise may lead to treatment strategies for obesity, diabetes, and associated metabolic sequelae.

Exercise increases energy expenditure, reduces fat mass and can genetically alter adipose tissue [6–10]. Adipose tissues are highly plastic, capable of robust expansion and contraction depending on external cues such as exercise, nutrient availability and hormone levels [11, 12]. The flexibility of adipose tissue mass is derived from changes in the size of existing adipocytes and changes in the rate of new adipocyte formation, which derive from adipose progenitors/stem cells [12, 13]. Adipose progenitors reside in a therapeutically accessible perivascular niche where they proliferate and differentiate in response to various signals [13, 14]. For example, high-fat diet (HFD) stimulates adipose stem cells to differentiate into adipocytes [15]. In contrast, exercise is thought to reduce progenitor adipogenic potential [16]. Despite a role for adipose progenitors in adipose tissue homeostasis and environmental responses, how exercise alters adipose progenitor cell dynamics—proliferation, quantity, quiescence and differentiation—is not fully understood.

One tissue that is particularly responsive to exercise is skeletal muscle. Indeed, exercise training activates signaling and metabolic pathways in skeletal muscle that improve exercise performance and potentially protect against obesity [17]. Muscle responds to exercise by remodeling the content of myosin, the contractile unit of the muscle fiber, and increasing the number of mitochondria [18]. This remodeling leads to a switch in muscle fiber-type, preferentially expressing type I myofibers (slow twitch, aerobic) attuned to fatty acid oxidation rather than type II (fast twitch, anaerobic) glycolytic myofibers that are more susceptible to exercise fatigue [18–20]. This change alters energy substrate utilization, thereby reducing muscle fatigue and wasting. Evidence suggests that skeletal muscle remodeling improves systemic energy homeostasis through release of soluble factors that signal to distal tissues, such as the brain and adipose tissue [21]. Elucidation of the mechanisms through which exercise and skeletal muscle regulate adipose tissue mass/function may help combat obesity and associated metabolic dysfunctions.

Here we examined the effect of exercise and skeletal muscle fiber-type switching on the ability of adipose progenitor cells to proliferate and differentiate into mature adipocytes. We found that exercise induces adipose tissue remodeling: decreasing adipose progenitor proliferation and blunting adipogenic potential. We also show that adipose progenitors are responsive to not only exercise but to the levels of type I (slow) skeletal muscle fibers. Mechanistically, we provide evidence that secreted factors from type I myofibers reduces adipose progenitor differentiation. These data support a model where exercise induces skeletal muscle fiber-type switching, which alters adipose progenitor cell dynamics through systemic factors.

## Material and Methods

### Mice

The PPARγ-tTA, TRE-Cre, TRE-H2B-GFP, miR-499 Tg, MCK-Cre and *Sox6*^*fl/fl*^ mice were described previously and used to generate AdipoTrak or muscle-specific mouse lines [14, 22, 23]. Mice were fed either normal (4% fat, Teklad) or high fat (60%, Research Diets) chow and kept on normal 12-hour light/dark cycles with water and food provided *ad libitum*. Fat content was measured using a minispec mq10 NMR Analyzer (Bruker). For glucose tolerance tests (GTTs), 1.25mg glucose/1g mouse weight was injected intraperitoneally (IP) after a 5-hour fast and blood glucose levels were measured at the indicated intervals for up to two hours as described [24]. Veterinary care was provided by the Division of Comparative Medicine. All animals were maintained under the guidelines of the UT Southwestern Medical Center Animal Care and Use Committee according to current NIH guidelines. The animal research performed in this study was approved by the UTSW Medical Center Animal Care Use Committee.

### Metabolic Chamber Studies

Physical activity and energy expenditure were monitored using a combined indirect calorimetry system (TSE Systems GmbH, Bad Homburg, Germany) [25]. Mice were housed individually at room temperature (22°C) under an alternating 12-hour light/dark cycle. Mice were acclimated for five days in a chamber prior to data acquisition, which included heat generation, oxygen consumption and carbon dioxide production.

### Voluntary Wheel Running

Mice were individually housed in cages equipped with a running wheel and allowed to run *ad libitum*. Running was recorded using a Dataquest Acquisition and Analysis System (Data Sciences International) for up to two months. Mice were fed normal (4% fat, Teklad) chow and kept on normal 12-hour light/dark cycles with water and food provided *ad libitum* [26].

### RNA Extractions and Real-Time PCR

Total RNA from adipose tissue, muscle tissue and various cells was extracted using TRIzol (Invitrogen). Briefly, tissue and cells were disrupted in TRIzol and then chloroform was added to separate nucleic acids from protein. This was centrifuged, with the resultant aqueous layer being mixed with isopropanol to precipitate the RNA. The mixture is further centrifuged, with the RNA forming a pellet. This pellet is then washed in 75% EtOH, dried and resuspended in distilled water. The RNA is then DNaseI-treated for one hour at 37°C, with EDTA then being added to stop the reaction. To make cDNA, we incubated the RNA with random hexamers, dNTPs and Moloney Murine Leukemia Virus Reverse Transcriptase as described [24]. Gene expression was analyzed using 7500 Real-Time PCR System (Applied Biosystems) using SYBR Green Master Mix reagent (Applied Biosystems). The values for gene expression were normalized by beta-actin expression.

### Isolation of Adipose Progenitor Cells and Adipocytes

Floating adipocytes and stromal vascular fraction (SVF) cells were isolated from explanted adipose depots as described [14]. Briefly, SVF cells were isolated from pooled white adipose depots (inguinal and interscapular depots) that were explanted and minced into fine pieces. The adipose pieces were then digested in adipocyte isolation buffer (100mM HEPES pH 7.4, 120mM NaCl, 50mM KCl, 5mM glucose, 1mM CaCl_2_, 1.5% BSA) containing 1mg/ml of type I collagenase (Worthington) at 37°C for 2 hours. The suspension was then passed through a 210μm mesh to remove undigested clumps and debris. The effluent was centrifuged at 500g for 10 minutes, with the SVF pelleting to the bottom and adipocytes forming a top layer. The adipocytes were removed from the top layer for either RNA or nuclei isolation. The SVF pellet was then resuspended in 155mM NH_4_Cl erythrocyte lysis buffer, and filtered through 30μm mesh. This was then centrifuged at 500g for 10 minutes and the pellet washed with PBS. The resultant isolated cells were subjected to FACS, RNA isolation, or plated in cell culture vessels.

### Adipocyte Nuclei Isolation

This procedure was performed as previously described [14]. Briefly, the adipocytes were isolated as described above and then resuspended in cold nuclei extraction buffer (320mM sucrose, 5mM MgCl_2_, 10mM HEPES, 1% Triton-X at pH7.4). The cells were vortexed gently for 10 seconds and incubated on ice for 15 minutes. Nuclei were pelleted at 2000xg, and washed twice with nuclei wash buffer (320mM sucrose, 5mM MgCl_2_, 10mM HEPES at pH7.4). Nuclei were then either prepared for FACS analysis or stained for BrdU.

### BrdU Staining

BrdU was either given in drinking water (0.05% BrdU, 0.1% Splenda) for two or seven days prior to analysis or injected intraperitoneally (50mg/kg body weight) and examined two hours later. For *in vitro* studies, BrdU was given 12 hours prior to analysis at a final concentration of 10μM. BrdU was analyzed using a BrdU staining kit (BD Biosciences) following manufacturer’s protocol. Briefly, cells or nuclei were fixed, permeabilized, DNase-treated and stained with anti-BrdU antibody. The cells/nuclei were stained with APC-conjugated secondary antibody, then with propidium iodide (PI) for DNA and analyzed using FACS. PI staining was used to distinguish cells from cell debris.

### SVF Culture

The SVF cells were cultured in Dulbecco's Modified Eagle Media (DMEM) with 10% FBS, 100units/ml penicillin and 100μg/ml streptomycin. For adipogenic induction, confluent wells were maintained in growth media supplemented with 1μg/ml insulin for at least three days. Oil Red O (0.5% in isopropanol) staining was performed as described [27]. Briefly, cells were fixed in 4% paraformaldehyde, washed, and then incubated in Oil Red O working solution (four parts water mixed with six parts Oil Red O) for one hour at room temperature. Cells are then washed and analyzed through light microscopy.

### Conditioned Media

C2C12 cells (purchased from ATCC) were incubated in growth medium (DMEM, 10% FBS, 100units/ml penicillin and 100μg/ml streptomycin). When confluent, C2C12 cells were placed in differentiation media (DMEM, 2% horse serum, 100units/ml penicillin and 100μg/ml streptomycin) and allowed to differentiate for 3 days. Conditioned media was collected from undifferentiated cells (myoblast conditioned media) and differentiated cells (myotube conditioned media), spun at 1500g to remove dead cells, supplemented with 1μg/mL insulin, and incubated on either SVF or 3T3-L1 cells (purchased from ATCC) until time of analysis. For the *ex vivo* muscle conditioned media, skeletal muscle was harvested from wild-type or mutant mice and placed on a Matrigel-coated 12-well plate. Growth media was then added to each well and conditioned media was collected two days after explant. Rspo3 and Fndc5 conditioned media was generated by transfecting Cos cells with pcDNA3.1-empty, pcDNA3.1-Rspo3 or pcDNA3.1-Fndc5, and collecting media 24 hours after transfection. Rspo3 recombinant protein was purchased from R&D Systems.

### Microarray Analysis

Triplicate samples of pooled total RNA from skeletal muscle were analyzed on an Illumina MouseWG-6 v2.0 BeadChip as described previously [22]. Relative fold-change was calculated by comparison of signal intensities. Fold-changes greater or less than two were subject to gene ontology analysis (Ingenuity Systems) with a statistical threshold of P = 0.01.

## Results

### Increased Caloric Intake Alters Adipose Progenitor Activity

To examine the response of adipose progenitor cells to physiological stimuli, we generated adipose-lineage GFP reporter mice (AdipoTrak-GFP) as previously reported [14, 28, 29]. Briefly, we crossed the PPARγ-tTA mouse line to the tet-response element (TRE)-H2B-GFP mouse strain to create PPARγ-tTA; TRE-H2B-GFP (AdipoTrak-GFP) mice. In this model, H2B-GFP is expressed in cells that express PPARγ, including both mature adipocytes and adipose progenitor cells. The production of H2B-GFP can be controlled with doxycycline (dox), where dox prevents the AdipoTrak expressed tTA from binding to the tet-response element, thus inhibiting H2B-GFP transcription. H2B-GFP is incorporated into chromatin during DNA replication, and therefore can be used as a proliferative indicator. Although H2B-GFP is stable in the nucleosome in a non-proliferative state, during proliferation the existing H2B-GFP is divided amongst its daughter cells. Thus, dox treatment prevents new H2B-GFP expression, which decreases GFP intensity of proliferative cells compared to cells that do not divide (S1 Fig).

We, and others, have shown that during normal adipose homeostasis mature adipocytes undergo apoptosis and get replaced by new adipocytes derived from the perivascular progenitor pool [12, 14, 30, 31]. Several studies indicate that high fat diet (60% fat, HFD) can increase the proliferation of the adipose depot stromal vascular fraction (SVF) that contains adipose stem cells/progenitors as well as various other cell types including endothelial cells and hematocytes [15, 32–37]. To investigate whether HFD alters adipose progenitor dynamics in our model, we provided AdipoTrak-GFP mice with HFD for eight weeks in the presence or absence of dox, and examined the persistence of GFP in both subcutaneous and visceral adipose depots. We analyzed the following groups of AdipoTrak-GFP mice: normal chow without dox (NC), normal chow with dox (NC+Dox), HFD without dox (HFD) and HFD with dox (HFD+Dox). As expected, the mice fed with HFD for two months exhibited increased body fat percentage and adipose depot mass compared to NC-fed mice, and the addition of doxycycline did not have a significant effect on these parameters (S2A and S2B Fig).

**Fig 1:**
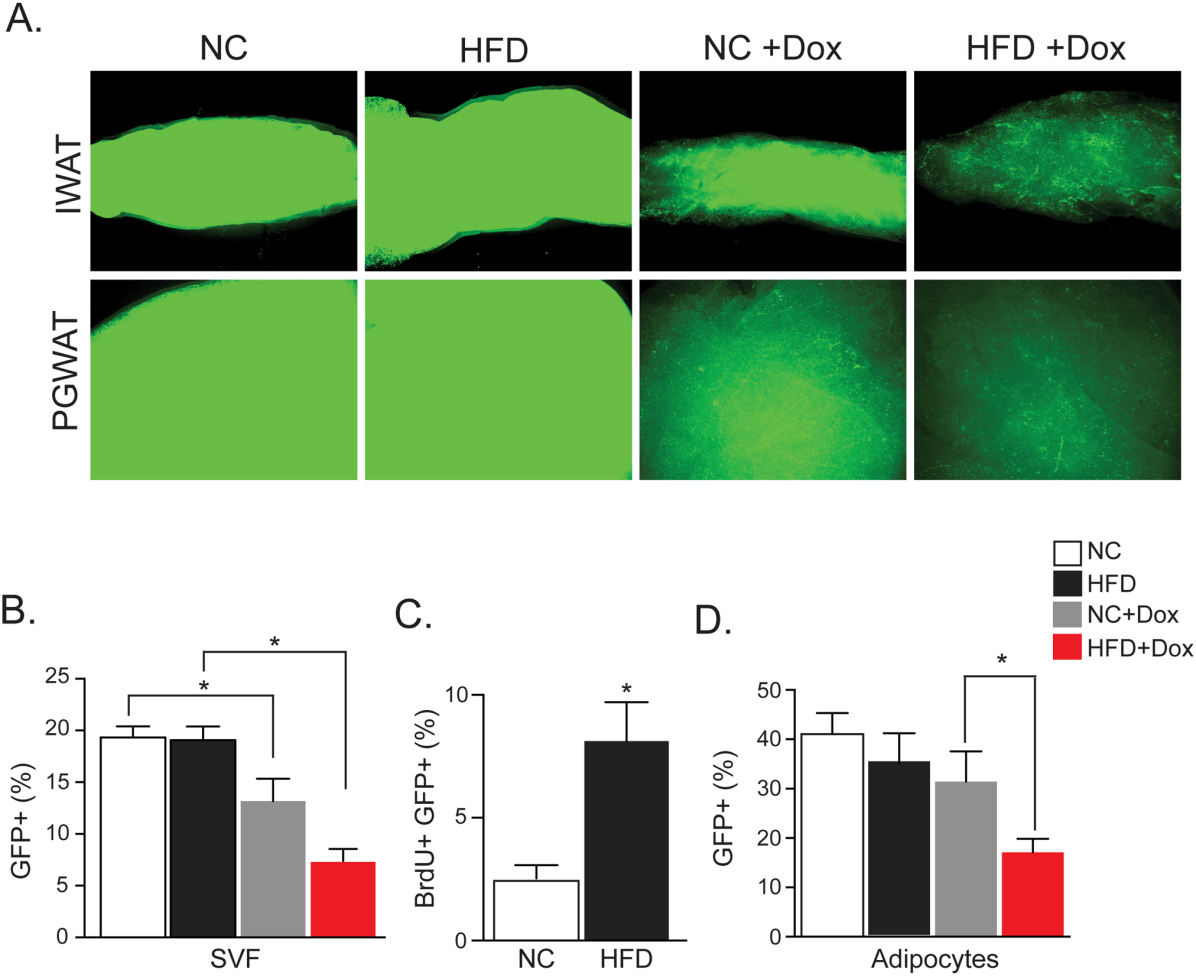
High Fat Diet Induces Adipose Progenitor Proliferation and Differentiation. (A) Fluorescent microscopy of inguinal (IWAT) and perigonadal (PGWAT) white adipose depots after two months of indicated treatment (NC: Normal chow diet, HFD: High fat diet). Two-second exposure used in order to visualize GFP in groups that received dox (+Dox). (B) Percentage of GFP-positive (GFP+) cells in the subcutaneous stromal vascular fraction (SVF) of mice two months after indicated treatments. (C) Percentage of GFP+ cells that are BrdU-positive (BrdU+) in the subcutaneous SVF of mice two months after indicated treatments. BrdU was given in the drinking water for two days prior to analysis. (D) Percentage of GFP+ nuclei in adipocyte fraction isolated from subcutaneous fat two months after indicated treatment. Representative studies on P120 males; n ≥ 12 per cohort, repeated ≥ 3 cohorts. Error bars indicate SEM. Statistical significance assessed by two-tailed Student’s t-test. *p<0.05.

We next examined adipose depot explants by whole-mount microscopy for expression and intensity of GFP. Dox treatment decreased H2B-GFP expression even in the normal chow state, further supporting the notion that adipocytes turnover during homeostasis (Fig 1A). We observed a greater reduction of GFP in whole depots from HFD-fed dox-treated mice, suggesting that HFD increased progenitor proliferation and adipocyte turnover (Fig 1A). To determine if GFP is altered in adipose progenitors or mature adipocytes after dox treatment, we isolated adipocytes and SVF from subcutaneous depots (inguinal and interscapular) from each of the four groups of mice. The percentage of cells from the progenitor population was then determined by FACS analysis. While dox treatment decreased the percentage of GFP-positive (GFP+) progenitor cells found in the SVF of mice fed normal chow, the HFD stimulus further diluted the GFP+ pool (Fig 1B). These data suggest that HFD induces proliferation and differentiation of adipose progenitors.

To test the cycling characteristics of adipose progenitors during HFD, we administered BrdU in the drinking water of normal chow or HFD AdipoTrak mice two days prior to sacrifice. FACS analyses of BrdU+ GFP+ progenitors revealed that HFD increased adipose progenitor proliferation (Fig 1C). We also analyzed GFP expression in the adipocyte fraction and found a significant decrease of GFP+ adipocytes in HFD+Dox mice, which indicates that HFD increases production/turnover of mature adipocytes (Fig 1D). Together, these studies are consistent with the idea that HFD feeding increases adipocyte turnover and activity of the progenitor population, and highlights the utility of using the AdipoTrak system to study progenitor and adipocyte regulation.

### Exercise Reduces Adipose Progenitor Function

**Fig 2:**
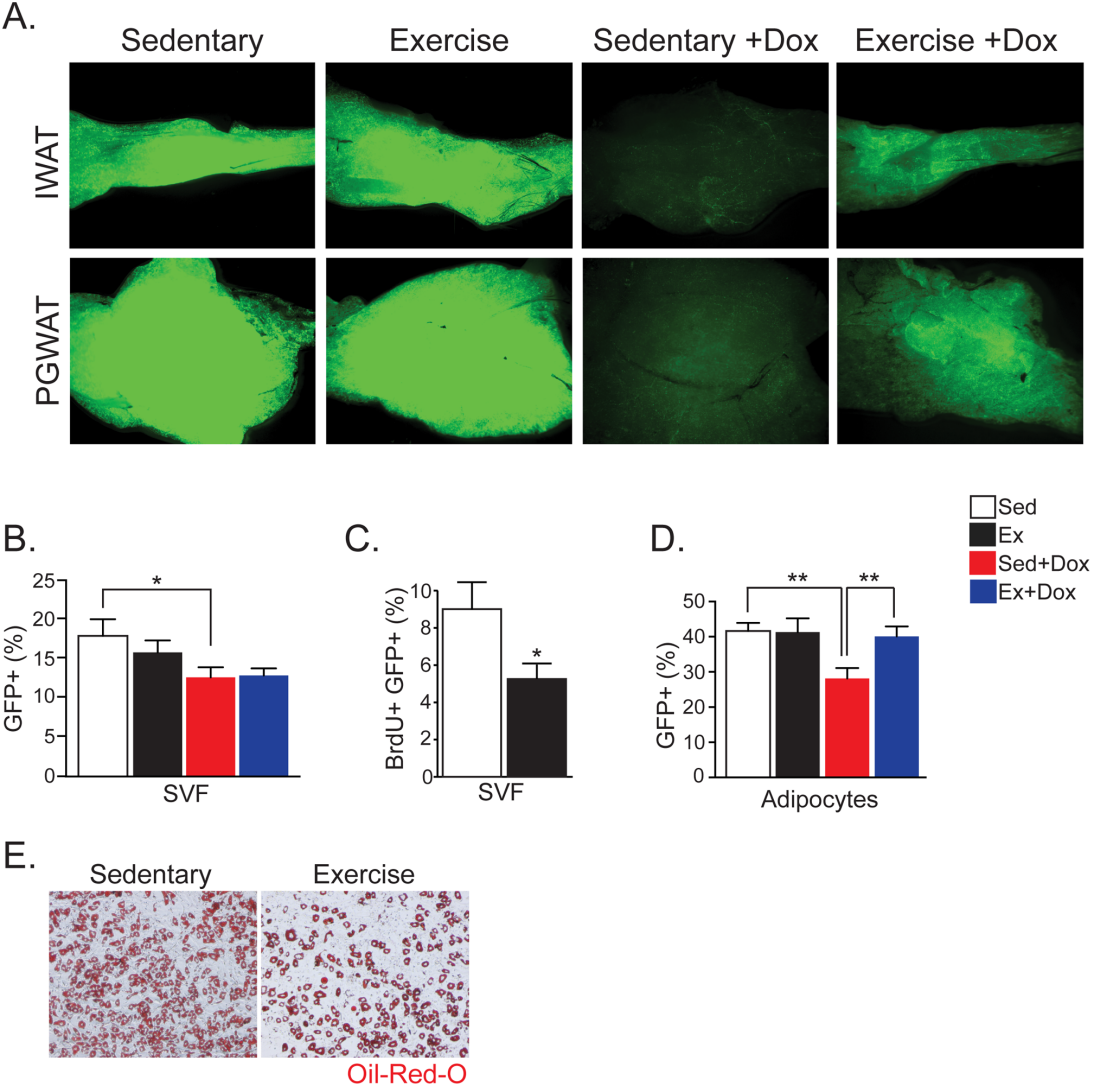
Exercise Inhibits Adipose Progenitor Proliferation and Differentiation. (A) Fluorescent microscopy of inguinal (IWAT) and perigonadal (PGWAT) white adipose depots after two months of indicated treatment. Two-second exposure used in order to visualize GFP in groups that received Dox (+Dox). (B) Percentage of GFP-positive (GFP+) cells in the subcutaneous stromal vascular fraction (SVF) of mice two months after indicated treatments (Sed: sedentary, Ex: exercise). (C) Percentage of GFP+ cells that are BrdU-positive (BrdU+) in the subcutaneous SVF of mice two months after indicated treatments. BrdU was given in the drinking water for two days prior to analysis. (D) Percentage of GFP+ nuclei in adipocyte fraction isolated from subcutaneous fat depots two months after indicated treatment. (E) Oil Red O staining of SVF from subcutaneous fat plated two months after indicated treatment. Cells were plated to confluency and stained four days post insulin induction. Representative studies on P120 males; n ≥ 12 per cohort, repeated ≥ 3 cohorts. Error bars indicate SEM. Statistical significance assessed by two-tailed Student’s t-test. *p<0.05, **p<0.01.

We next exploited the AdipoTrak system to interrogate the effect of endurance exercise (wheel running) on the adipose lineage. To that end, we randomized two-month old AdipoTrak-GFP siblings into four groups: sedentary alone (Sed), sedentary plus dox (Sed+Dox), exercise alone (Ex) and exercise plus dox (Ex+Dox). Over 8 weeks, each group was given normal chow and those in the exercise groups were subjected to eight weeks of voluntary wheel running, after which the adipose tissue was analyzed for progenitor activity using similar assays to those described above. As expected, two months of *ad libitum* wheel running decreased body fat percentage, adipose depot size and mass (S3A and S3B Fig). The administration of dox water did not alter exercise or its effect on body fat (S3A–S3C Fig). To characterize the response of adipose tissue to exercise we evaluated adipose progenitor proliferation, differentiation, and mature adipocyte turnover by examining adipose depot explants for GFP expression. We found that exercised adipose depots, Ex+Dox, exhibited significantly increased GFP intensity compared to sedentary mice, Sed+Dox, which is the opposite to the HFD-feeding effect. These data support the notion that exercise alters adipose homeostasis either through a decrease in progenitor activity or a reduction in adipocyte turnover (Fig 2A).

To determine the effect of exercise on adipose progenitors, we isolated the progenitor-containing SVF from all four groups, and subjected the cells to flow cytometry for GFP. Surprisingly, this analysis revealed a similar reduction of GFP+ cells in both sedentary and exercise dox-treated samples (Fig 2B). While GFP levels in the SVF were not different, we did detect a reduction in proliferation, as assessed by BrdU incorporation, of the GFP+ progenitor pool during the exercise regimen (Fig 2C). This discrepancy is most likely secondary to the cellular dynamics of the AdipoTrak-GFP system, which produces GFP-negative (GFP-) cells from multiple rounds of division of GFP+ cells, as well as new GFP+ cells from those initial rounds of division, although at a lesser intensity (S1 Fig). In a stable population (i.e. exercise compared to sedentary), decreased proliferation will produce less GFP- cells but also less new, lower-intensity, GFP+ cells. Therefore, decreased proliferation in the AdipoTrak-GFP system may not produce noticeable changes in the total number of GFP+ cells compared to control as production of both new GFP+ and GFP- cells are decreased.

The increased GFP intensity noted in adipose depots of the Ex+Dox mice compared to Sed+Dox (Fig 2A) could be due to reduced adipocyte turnover that necessitates fewer new cells in a time of increased energy expenditure. To test this, we analyzed the turnover of mature adipocytes during exercise and the ability for progenitor cells to differentiate in culture. Adipocytes from Ex+Dox mice exhibited a higher percentage of GFP+ nuclei compared to Sed+Dox mice suggesting a decrease in adipocyte production/turnover (Fig 2D). To examine differentiation, we removed the SVF from exercised and sedentary subcutaneous fat depots and induced them to undergo adipogenesis *in vitro*. Oil Red O analysis indicated that the SVF from sedentary mice differentiated more efficiently compared to SVF from exercised mice supporting *in vivo* adipocyte turnover data (Fig 2E). Taken together, the data support the possibility that exercise decreases adipose progenitor proliferation and differentiation.

### Adipose Progenitors Respond to Skeletal Muscle

Multiple organ systems alter their physiological properties in response to endurance exercise. One prominent tissue that is regulated by exercise is skeletal muscle, where training leads to a switch in the contractile proteins and metabolic properties in the myofibers [17–20]. For example, there is a fast to slow myofiber conversion observed with endurance exercise, characterized by an increase in slow myosin content in muscle fibers and a concomitant decrease in fast myosin [17–20]. To investigate the possibility that the shift to slow muscle fibers after endurance training might contribute to the observed adipose progenitor phenotype, we utilized two genetic mouse models that harbor predominantly slow muscle fibers. The first model expresses miR-499 under the control of a muscle creatine kinase (MCK) promoter (miR-499 Tg) [23]. The second genetic exercise model harbors a conditional Sox6 allele, *Sox6*^*fl/fl*^, and a muscle-specific MCK-Cre allele, which results in loss of Sox6 in muscle fibers (Sox6 mKO) [22]. We first analyzed these slow fiber mice for adipose or metabolic phenotypes on normal chow. Body fat percentage, adipose depot weights, glucose tolerance, and energy utilization were unchanged compared to controls in both genetic exercise cohorts (S4A–S4D and S5A–S5C Figs). These data indicate that the slow fiber mutant mice are metabolically normal at baseline.

**Fig 3:**
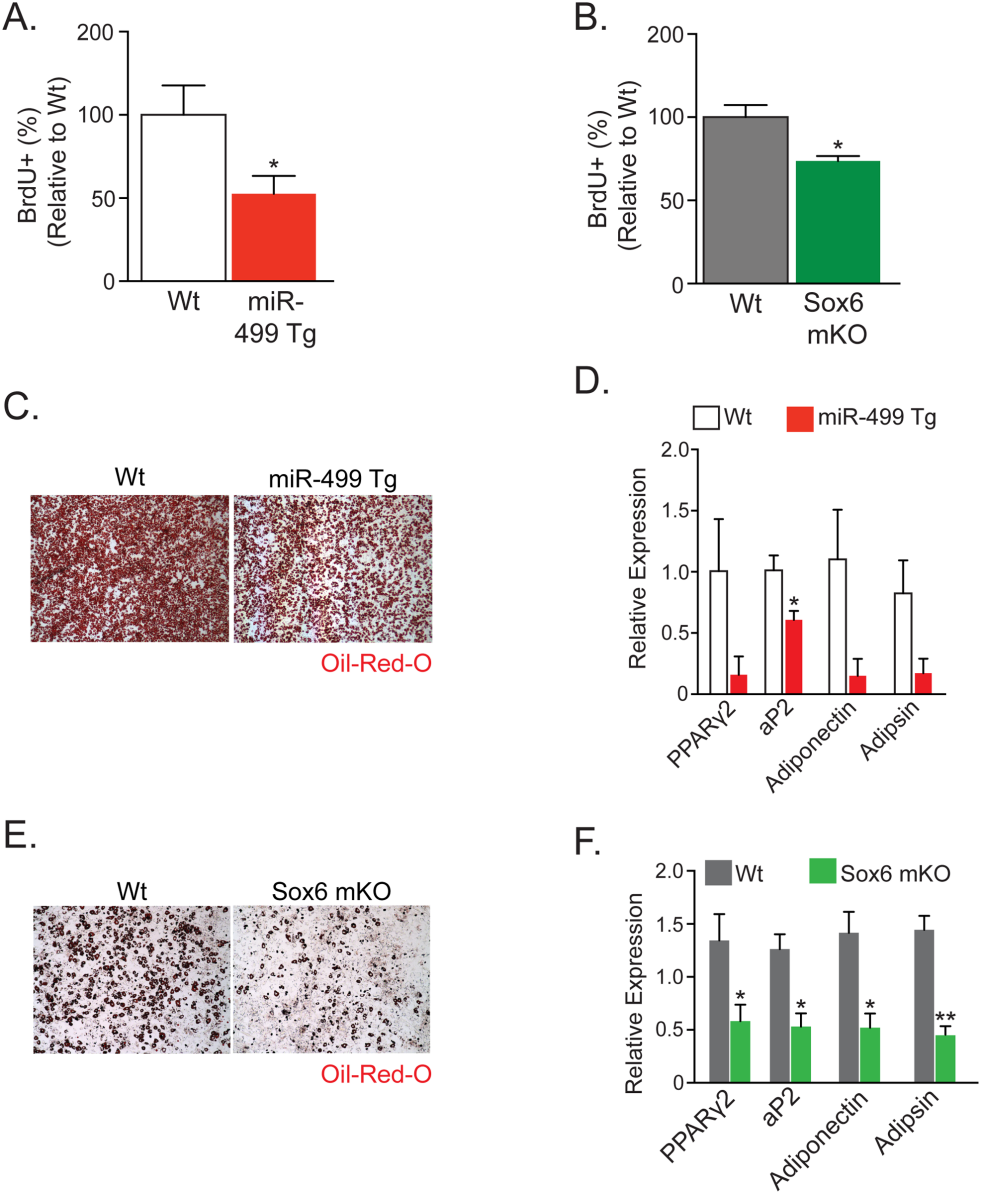
Decreased Proliferation and Adipogenic Gene Profile of Stromal Vascular Fraction in Mice with Slow Muscle Fiber-Type Predominance. (A) Percentage of BrdU-positive (BrdU+) cells within total stromal vascular fraction (SVF) of wild-type (Wt) and miR-499 Tg subcutaneous depots. BrdU was given as a two-hour pulse injected intraperitoneally (IP). (B) Percentage of BrdU+ cells within total SVF of Wt and Sox6 mKO subcutaneous depots. BrdU was given as a two-hour pulse injected IP. (C) Oil Red O staining of plated subcutaneous SVF after four days of insulin induction of both Wt and mir-499 Tg mice. (D) qPCR of adipogenic markers from adipogenically-induced SV cells isolated from subcutaneous adipose depots from Wt and mir-499 Tg mice. (E) Oil Red O staining of plated subcutaneous SVF after four days of insulin induction of both Wt and Sox6 mKO mice. (F) qPCR of adipogenic markers from adipogenically-induced SVF isolated from subcutaneous adipose depots from Wt and Sox6 mKO mice. Representative studies on P60 females; n ≥ 8 per cohort, repeated ≥ 3 cohorts. Error bars indicate SEM. Statistical significance assessed by two-tailed Student’s t-test. *p<0.05, **p<0.01.

We next evaluated the properties of adipose progenitors in both miR-499 Tg and Sox6 mKO mice. We administered BrdU and examined the SVF to determine whether the exercise-induced changes in adipose progenitor cells might also be present in mice with more type I (slow) myofibers. Consistent with that notion, we found significantly lower BrdU levels in both miR-499 Tg and Sox6 mKO mice compared to controls, showing reduced proliferation potential of SVF from subcutaneous fat pads (Fig 3A and 3B). To test differentiation potential, we isolated subcutaneous SVF from both types of slow fiber mice and control siblings and cultured them in adipogenic conditions. The SVF from both miR-499 Tg and Sox6 mKO displayed inhibited differentiation compared to controls, based upon Oil Red O staining and qPCR analyses of adipogenic gene expression (Fig 3C–3F). Taken together, our data support the hypothesis that the models of slow skeletal muscle result in an altered adipose progenitor compartment, similar to what was noted in our AdipoTrak-exercise model (Fig 2C and 2E).

### Slow Fibers Reduce Adiposity during HFD Feeding

**Fig 4:**
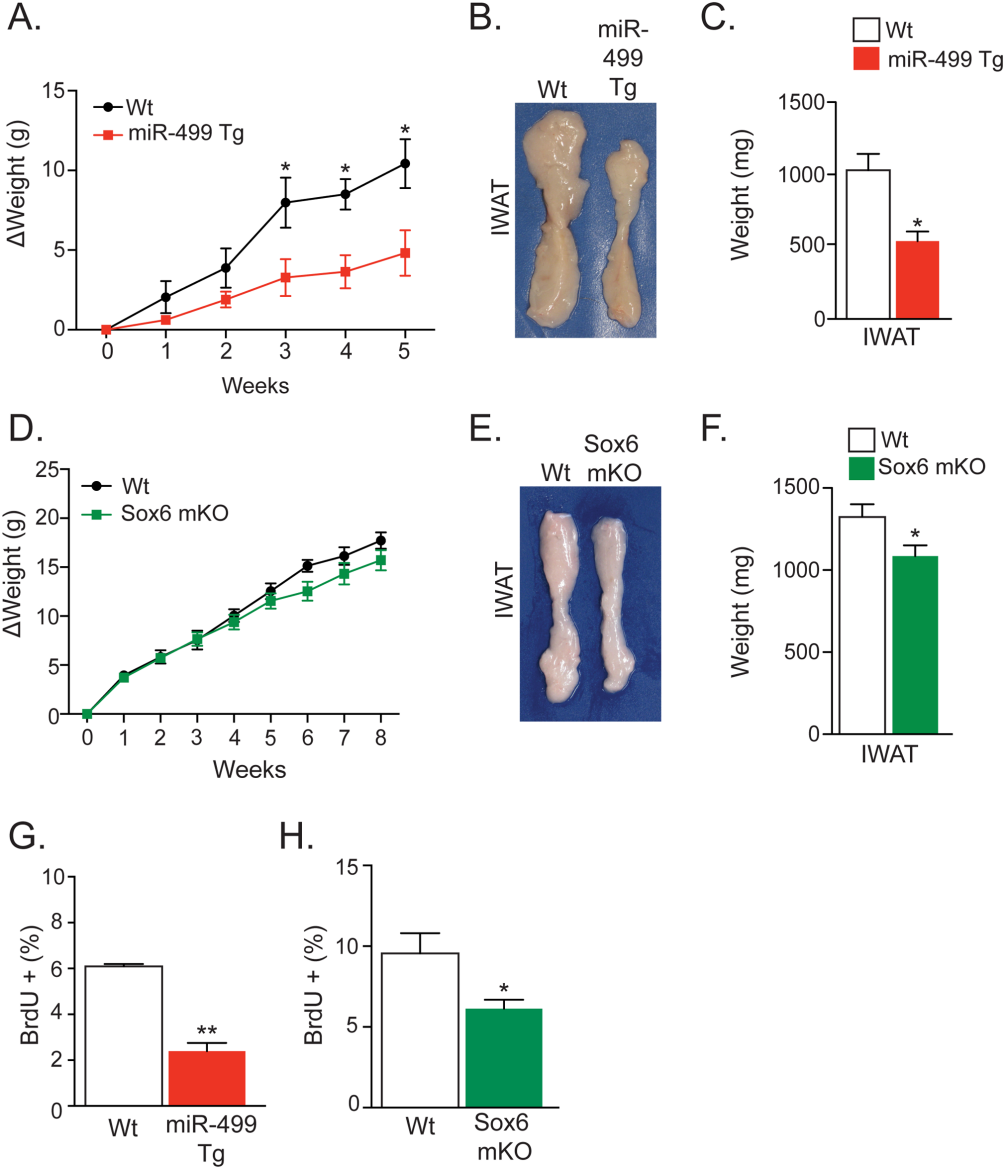
Slow Fiber Mutant Mice Are Resistant to Diet-Induced Weight Gain. (A) Weekly weight change during high fat diet (HFD) feeding of wild-type (Wt) and miR-499 Tg female mice. (B and C) Inguinal (IWAT) fat pads were removed from Wt and miR-499 Tg female mice after 5 weeks of HFD. Representative explants of IWAT are shown in (B) with total weights of IWAT shown in (C). (D) Weekly weight change during HFD feeding of Wt and Sox6 mKO male mice. (E and F) Inguinal (IWAT) fat pads were removed from Wt and Sox6 mKO male mice after 8 weeks of HFD. Representative explants of IWAT are shown in (E) with total weights of IWAT shown in (F). (G and H) Percentage of BrdU-positive (BrdU+) adipocyte nuclei obtained from subcutaneous fat depots of Wt mice compared to female miR-499 Tg (G) and male Sox6 mKO mice (H). BrdU given during last week of HFD treatment in drinking water. Representative studies on P100-120 mice; n ≥ 8 per cohort, repeated ≥ 3 cohorts. Error bars indicate SEM. Statistical significance assessed by two-tailed Student’s t-test. *p<0.05, **p<0.01.

To investigate whether the observed changes in adipose progenitor cells might have physiological consequences, we fed miR-499 Tg and Sox6 mKO mice HFD for two months. We turned to this provocative challenge, which stimulates proliferation and differentiation, as it might elicit a metabolically relevant phenotype in the type I fiber mice. Consistent with that notion, we found that miR-499 Tg mice exhibited less weight gain while receiving HFD (Fig 4A). The inguinal (IWAT) depot size was smaller in miR-499 Tg mice compared to Wt controls (Fig 4B and 4C). We observed similar results with HFD-fed Sox6 mKO mice (Fig 4D–4F), which are consistent with observations in other genetically-modified mice that contain more slow myofibers [38, 39].

Our data suggest that adipose progenitors in slow fiber mice are recruited to mature adipocytes at a lower frequency compared to Wt mice; there is decreased subcutaneous adipose depot size after HFD (Fig 4C and 4F) and there appeared to be blunted *in vitro* differentiation of SVF isolated from subcutaneous depots (Fig 3C–3F). To attempt to extend the cell culture findings to intact animals, we administered BrdU to miR-499 Tg and Sox6 mKO mice during the final week of the HFD regimen. In this experimental design, BrdU incorporates into cycling adipose progenitors, and detection of BrdU in the floated adipocyte compartment indicates differentiation of the progenitor into mature adipocytes. To evaluate the level of BrdU in adipocytes, we isolated adipocyte nuclei and quantitated BrdU using flow cytometry [14, 28]. We found that miR-499 Tg and Sox6 mKO adipocyte nuclei had a significant decrease in BrdU incorporation (Fig 4G and 4H). These data indicate that mice with more type I (slow) skeletal muscle are protected from HFD-induced weight gain potentially due to decreased adipogenic potential of adipose progenitors.

### Muscle Conditioned Media Reduces SVF Proliferation and Differentiation

**Fig 5:**
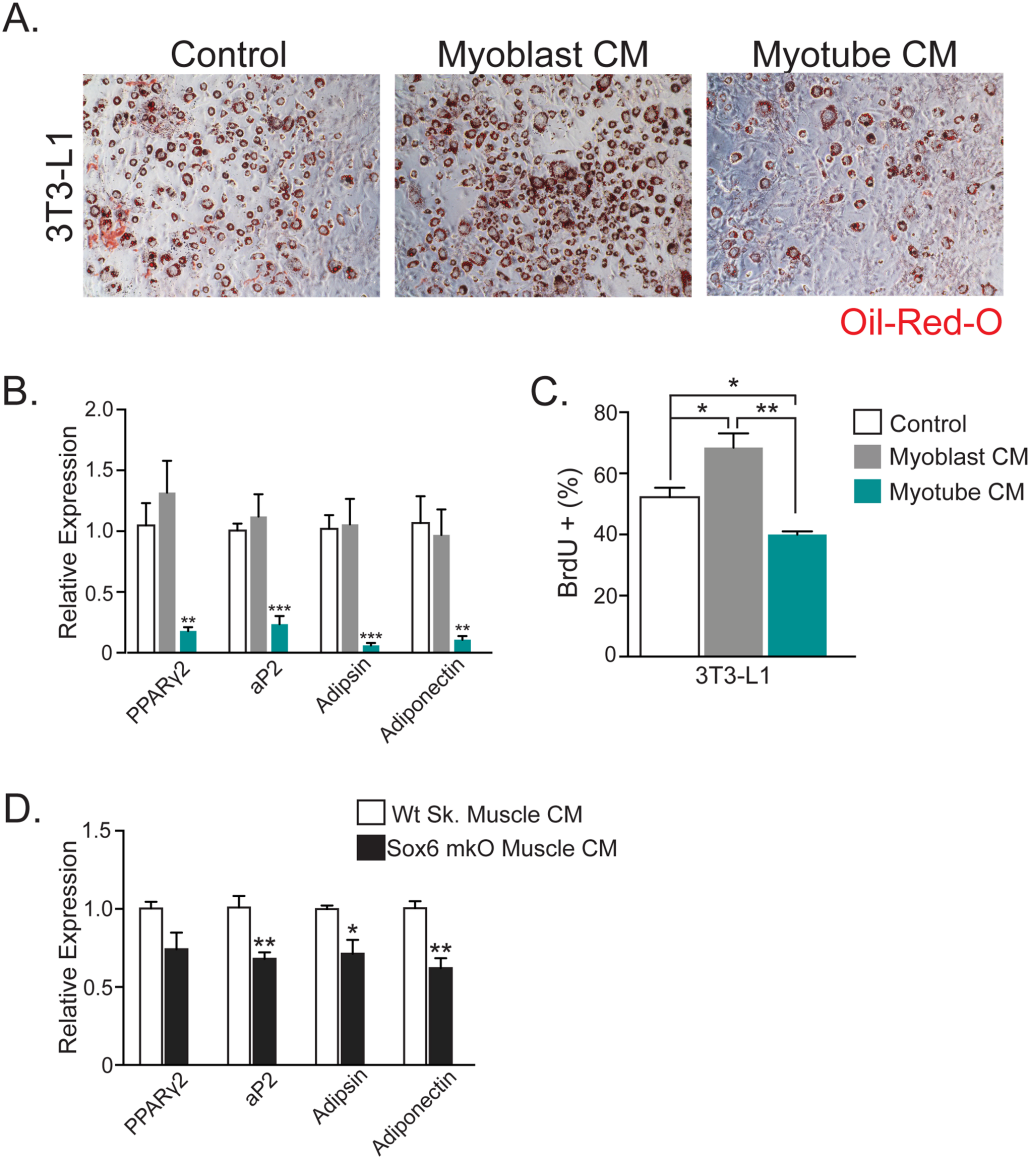
Conditioned Media From Mature Muscle Inhibits Adipogenesis. (A) Oil Red O staining of 3T3-L1 cells after four days of adipogenic induction using control media (2%HS, control), conditioned media from uninduced C2C12 cells (myoblast CM) or conditioned media from induced, mature C2C12 cells (myotube CM), all supplemented with insulin. C2C12 cells were grown in 2% HS. HS: horse serum, CM: conditioned media. (B) Expression of adipogenic markers from 3T3-L1 cells by qPCR after four days of adipogenic induction using control, myoblast CM or myotube CM. (C) Percentage of BrdU-positive (BrdU+) 3T3-L1 cells incubated in control, myoblast CM or myotube CM for 12 hours during clonal expansion. (D) Expression of adipogenic markers from stromal vascular fraction (SVF) of subcutaneous fat by qPCR after four days of adipogenic induction using conditioned media from wild-type (Wt) or Sox6 mKO gastrocnemius muscle with supplemented insulin. n ≥ 6 per cohort, repeated ≥ 3 cohorts. Error bars indicate SEM. Statistical significance assessed by two-tailed Student’s t-test. *p<0.05, **p<0.01, ***p<0.001.

To assess whether the exercise and type I (slow) fiber models might mediate the observed adipose progenitor phenotypes via secreted humoral factors, we turned to *in vitro* systems. For this, we used conditioned media from C2C12 proliferating myoblasts and media from C2C12 myotubes, the latter is a model of terminally-differentiated muscle, to treat the widely used 3T3-L1 adipogenic cell line. We assessed the proliferative and differentiation response of 3T3-L1 cells after exposure to conditioned media from muscle cells. Compared to control and myoblast conditioned media, myotube conditioned media significantly reduced the differentiation potential of 3T3-L1 cells as assessed by Oil-Red-O staining (Fig 5A). qPCR analyses also revealed a reduction of adipogenic gene expression after exposure to myotube-conditioned media (Fig 5B). To evaluate proliferation of 3T3-L1 cells after treatment with muscle conditioned media, we incubated the cells with BrdU for 12 hours during the expansion phase (clonal expansion) of adipogenic differentiation. Myotube conditioned media also reduced proliferation of 3T3-L1 cells, suggesting the presence of factors from muscle that alter both adipose progenitor proliferation and differentiation (Fig 5C).

We next evaluated the hypothesis that type I muscle fibers exert a greater effect on SVF differentiation potential compared to wild-type skeletal muscle. Here, we dissected muscles from wild-type or Sox6 mKO mice, explanted these muscles in matrigel, and incubated them in standard cell culture media. Conditioned media from these explants were then incubated with SVF from wild-type mice, which were then adipogenically-differentiated and analyzed by qPCR for adipogenic gene expression. Conditioned media from Sox6 mKO mice decreased adipocyte gene expression during SVF differentiation (Fig 5D). Together these data are consistent with a model in which skeletal muscle regulates adipose progenitor function through secreted factors.

### R-spondin 3 Reduces Adipogenesis *In Vitro*

**Fig 6:**
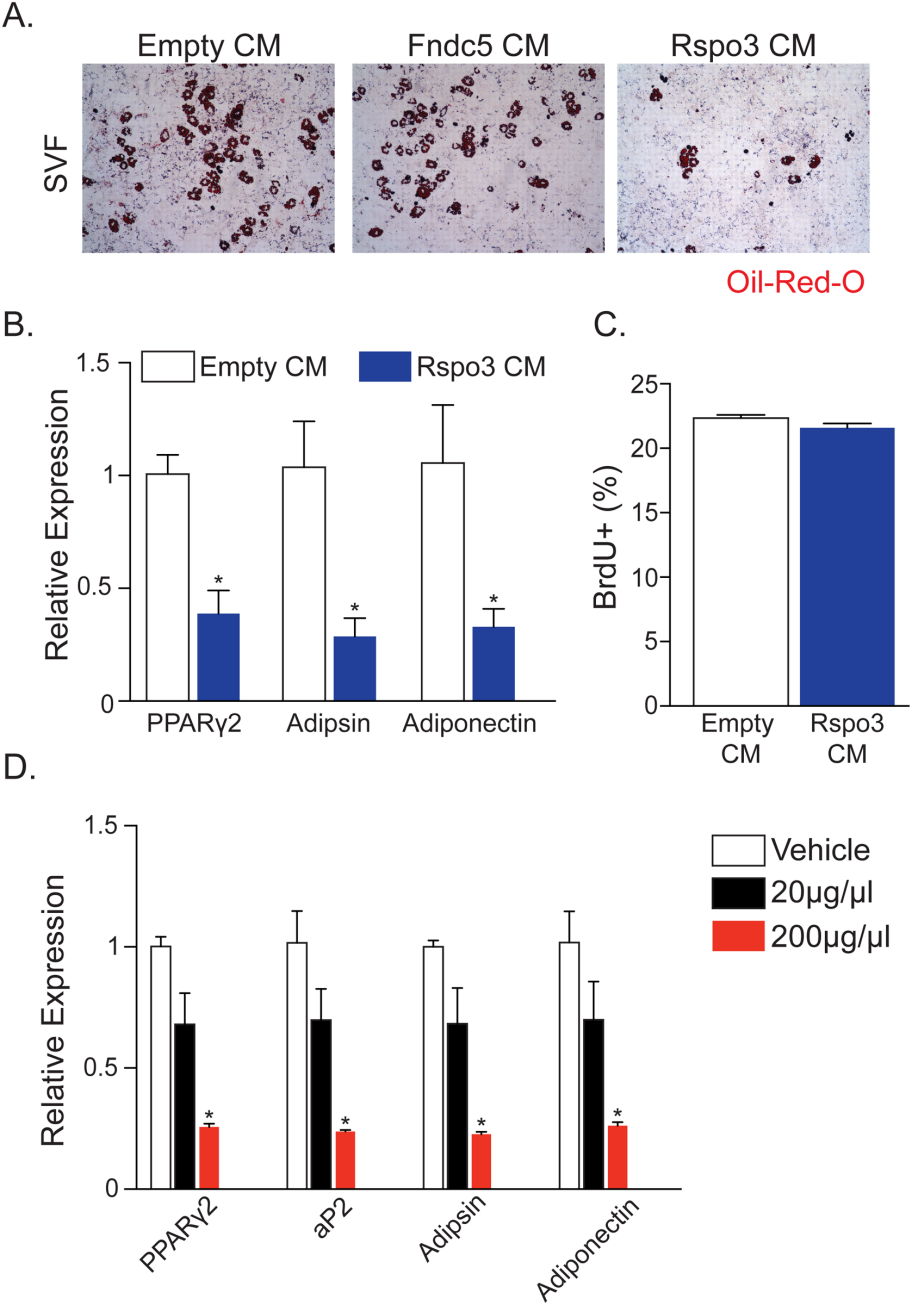
Rspo3 Inhibits Adipogenesis, But Not Proliferation, of Stromal Vascular Cells. (A) Oil Red O staining of stromal vascular fraction (SVF) cells after four days of adipogenic induction using control media (10% FBS with empty vector, empty CM), 10% FBS conditioned with Fndc5 (Fndc5 CM) or 10% FBS conditioned with Rspo3 (Rspo3 CM), all supplemented with insulin. CM: conditioned media. (B) qPCR of adipogenic markers from SVF of subcutaneous fat after four days of adipogenic induction using empty CM or Rspo3 CM. (C) Percentage of BrdU-positive (BrdU+) cells in SVF cells incubated in empty CM or Rspo3 CM for 12 hours. (D) qPCR of adipogenic markers from SVF of subcutaneous fat after four days of adipogenic induction using 10% FBS or 10% FBS conditioned with either 20μg/μL or 200μg/μL of recombinant Rspo3 protein. n ≥ 6 per cohort, repeated ≥ 3 cohorts. Error bars indicate SEM. Statistical significance assessed by two-tailed Student’s t-test. *p<0.05.

To attempt to identify factors from skeletal muscle that might underlie communication between skeletal muscle and adipose tissue, we interrogated microarray data from both miR-499 Tg and Sox6 mKO muscles [22]. We sorted this list of genes for those that contain a signal peptide, an indication of secretion in the absence of transmembrane domains. This analysis resulted in 12 genes encoding potentially secreted proteins that were upregulated in both slow fiber models (S1 Table). From this list we focused on two factors, Fndc5, also known as irisin, and R-spondin 3 (Rspo3). We chose Fndc5 because of its identified role as an exercise-induced factor that causes a browning of white adipose tissue [40]. Rspo3 was also an attractive candidate because it exhibits Wnt-activating properties [41], a pathway known to decrease adipogenesis [29, 42]. To test whether Fndc5 or Rspo3 regulates the adipogenic potential of the adipose progenitor compartment, we generated conditioned media with increased expression of Fndc5 or Rspo3 by transfecting Cos cells with empty vector or plasmids that express either Fndc5 or Rspo3. We collected conditioned media (CM) and added that to cultured SVF cells isolated from wild-type adipose depots. Although Fndc5 CM did not alter SVF adipogenesis, Rspo3-conditioned media significantly reduced adipogenesis, based upon Oil Red O staining and adipocyte gene expression (Fig 6A and 6B). However, Rspo3 did not affect SVF proliferation suggesting that Rspo3 specifically functions to inhibit differentiation (Fig 6C). We then incubated various concentrations of recombinant Rspo3 on SVF, assessed differentiation by qPCR, and discovered a robust anti-adipogenic activity for Rspo3 at 200μg/μL (Fig 6D).

## Discussion

Exercise is a robust method to decrease fat mass and improve metabolic health. While effects of exercise on adiposity have been studied for decades [1, 6–8], whether or how exercise alters adipose progenitor cells remains incompletely understood. We reasoned that during periods of muscle contraction, when circulating nutrients are being provided by mature adipocytes, not stored by them, there would be no need for newly formed adipocytes and thus adipose progenitors may become quiescent. We used the previously described AdipoTrak-GFP system [14, 28, 29] to measure adipocyte differentiation and adipose progenitor proliferation after two months of wheel running. Strikingly, *in vivo* and *in vitro* adipogenesis, as well as *in vivo* progenitor proliferation, were all suppressed after endurance exercise.

Voluntary wheel running is not the only form of endurance exercise as multiple other techniques, including forced treadmill running, are also commonly employed. All of these forms of endurance exercise have similar effects on adipose tissue including decreased adiposity, decreased adipose tissue inflammation and altered adipocyte gene expression [9, 43–46]; glucose homeostasis appears to be improved more with voluntary wheel running when compared to forced treadmill running [43, 47, 48]. Further studies will need to investigate the effects different types of endurance exercise will have on the adipose progenitor population. Of note, it has been shown that treadmill running decreases adipogenesis in the stromal vascular fraction *in vitro* [16] and suggests decreased adipocyte turnover with reduced apoptosis of mature adipocytes compared to controls [46].

Since understanding the basis of mechanistic control of adipose progenitor function has basic and clinical ramifications, we examined other tissues that are altered by exercise and that could act on adipose in a non-autonomous manner. The endocrine response to exercise has been well studied and has shown alterations in the secretion of and response to many hormones known to affect adiposity, including thyroid hormone, growth hormone and insulin [43, 49–51]. One of the other main changes that occur with endurance exercise is a shift from type II (fast) to type I (slow) muscle fibers [18–20]. As muscle is an endocrine organ [21], we focused our investigation on this fiber-type switch.

To examine the effect of type I (slow) fibers on adiposity, we utilized two mouse strains (miR-499 Tg and Sox6 mKO) that have predominantly slow muscle fibers [22, 23]. Thus, we were able to focus on the muscle fiber-type switch that occurs with endurance exercise without the physical stress and other sequelae that occur during exercise itself. Although both strains showed normal metabolic phenotypes on a normal chow diet, total SVF cells showed decreased proliferation, while plated SVF displayed decreased adipogenesis. To see if these changes may alter responses of adipose tissue, we placed the slow fiber mutant mice on a high fat diet to provoke adipose tissue hypertrophy and hyperplasia. We found reduced weight gain and decreased subcutaneous depot size in our mutant models, similar to previous studies of other slow fiber models [38, 39].

These data suggest that type I (slow) skeletal muscle may regulate adipose progenitor function. To explore this notion, we exploited *in vitro* studies to examine a communicative link between muscle and adipose progenitors. In these assays we found that conditioned media from C2C12 myotubes, but not C2C12 myoblasts, suppressed both 3T3-L1 proliferation and adipogenesis. This was further supported by *ex vivo* assays in which wild-type SVF cells were cultured in conditioned media from explanted muscle tissue from either slow fiber mice or controls. In these experiments, the slow fiber mutant conditioned media repressed adipogenic gene expression when compared to wild-type conditioned media.

Multiple secreted factors have been identified from myocytes [21]; however, type I (slow) muscle fibers have not been the sole source of such searches. In our microarrays, we identified Rspo3 as a possible candidate to act on adipose progenitor cells: it is highly expressed in slow fiber myocytes, is secreted, and has Wnt-activating function [41], which has been shown previously to block adipogenesis [29, 42]. Rspo3, in a dose-dependent manner, reduced the expression of adipogenic gene in cell culture differentiation assays. While Rspo3 reduces adipogenesis *in vitro*, future work will require investigation of this possible signaling axis *in vivo*.

In summary, this work characterizes the response of adipose progenitor cells to HFD, exercise, and skeletal muscle. We provide evidence that exercise regulates the proliferation and differentiation potential of these cells. Further, our data support the notion that skeletal muscle can modify adipose progenitor activity, via secreted factors, which ultimately could lead to the development of novel treatment strategies for obesity and metabolic syndrome.

## Supporting Information

S1 Fig Design of AdipoTrak Experiments. In the AdipoTrak mice, H2B-GFP expression is controlled by doxycycline (dox). In the absence of dox, H2B-GFP is similarly expressed in all adipose progenitor cells, regardless of stimulus (top row). Once dox is added to the system, H2B-GFP production is inhibited, with remaining H2B-GFP found within chromatin. H2B is a stable histone not broken down during mitosis. Therefore, division of adipose progenitors produces cells with decreased GFP intensity, as H2B-GFP is split between daughter cells. This allows us to investigate the proliferative capacity of the progenitor cells with or without stimulus through dox, since less GFP equates to increased proliferation (bottom row).
(TIF)

S2 Fig Increased Adipose Mass in Mice on High Fat Diet. (A) Body fat percentage of mice on indicated treatment (NC: normal chow, HFD: high fat diet), either with dox (+Dox) or without dox. (B) (Left) Adipose depot weights of mice on indicated treatment (IWAT: inguinal white adipose tissue, ISCW: interscapular white adipose tissue, PGWAT: perigonadal white adipose tissue). (Right) Spleen weight of mice on indicated treatment. Representative studies on P120 males; n ≥ 16 per cohort, repeated ≥ 3 cohorts. Error bars indicate SEM. Statistical significance assessed by two-tailed Student’s t-test. *p<0.05, **p<0.01, ***p<0.001.
(TIF)

S3 Fig Decreased Adipose Mass in Mice on High Fat Diet. (A) Body fat percentage of mice on indicated treatment (Sed: sedentary, Ex: exercise), either with dox (+Dox) or without dox. (B) (Left) Adipose depot weights of mice on indicated treatment (IWAT: inguinal white adipose tissue, ISCW: interscapular white adipose tissue, PGWAT: perigonadal white adipose tissue). (Right) Spleen weight of mice on indicated treatment. (C) Rate of wheel revolutions (cycles/min) of indicated mice on running wheel. Representative studies on P120 males; n ≥ 16 per cohort, repeated ≥ 3 cohorts. Error bars indicate SEM. Statistical significance assessed by two-tailed Student’s t-test. *p<0.05, **p<0.01, ***p<0.001.
(TIF)

S4 Fig Slow Fiber Mutant Mice Maintain Normal Adipose Mass at Baseline. (A and B) Weight of inguinal fat depot (A) and total body fat content (B) of wild-type (Wt) and mir-499 Tg mice. (C and D) Weight of inguinal fat depot (C) and total body fat content (D) of Wt and Sox6 mKO mice. Representative studies on P60 females; n ≥ 8 per cohort, repeated ≥ 3 cohorts. Error bars indicate SEM.
(TIF)

S5 Fig Slow Fiber Mutant Mice Maintain Normal Blood Glucose Levels and Normal Oxygen Consumption Rates at Baseline. (A) Glucose tolerance test (GTT) of wild-type (Wt) and mir-499 Tg mice. (B) GTT of Wt and Sox6 mKO mice. (C) Oxygen consumption (VO_2_) of Wt and Sox6 mKO mice over a 24-hour period. Representative studies on P60 females; n ≥ 8 per cohort, repeated ≥ 3 cohorts. Error bars indicate SEM. Statistical significance assessed by two-tailed Student’s t-test.
(TIF)

S1 Table Genes Upregulated in Slow Fiber Mutant Mice That Encode Signal Peptides. Microarray analysis was performed on both mir-499 Tg and Sox6 mKO skeletal muscle compared to wild-type. Genes encoding a signal peptide with a greater than 2-fold expression in either mutant strain are shown.
(TIF)

## References

[1] Curioni CC, Lourenco PM. Long-term weight loss after diet and exercise: a systematic review. International journal of obesity. 2005;29(10):1168–74. 10.1038/sj.ijo.0803015 . 15925949

[2] Sodlerlund A, Fischer A, Johansson T. Physical activity, diet and behaviour modification in the treatment of overweight and obese adults: a systematic review. Perspectives in public health. 2009;129(3):132–42. . 19514637 10.1177/1757913908094805

[3] Lee IM, Shiroma EJ, Lobelo F, Puska P, Blair SN, Katzmarzyk PT, et al Effect of physical inactivity on major non-communicable diseases worldwide: an analysis of burden of disease and life expectancy. Lancet. 2012;380(9838):219–29. 10.1016/S0140-6736(12)61031-9 22818936 PMC3645500

[4] King NA, Horner K, Hills AP, Byrne NM, Wood RE, Bryant E, et al Exercise, appetite and weight management: understanding the compensatory responses in eating behaviour and how they contribute to variability in exercise-induced weight loss. British journal of sports medicine. 2012;46(5):315–22. 10.1136/bjsm.2010.082495 . 21596715

[5] Bauman AE, Reis RS, Sallis JF, Wells JC, Loos RJ, Martin BW, et al Correlates of physical activity: why are some people physically active and others not? Lancet. 2012;380(9838):258–71. 10.1016/S0140-6736(12)60735-1 . 22818938

[6] Poirier P, Despres JP. Exercise in weight management of obesity. Cardiology clinics. 2001;19(3):459–70. . 11570117 10.1016/s0733-8651(05)70229-0

[7] Arner P. Impact of exercise on adipose tissue metabolism in humans. International journal of obesity and related metabolic disorders: journal of the International Association for the Study of Obesity. 1995;19 Suppl 4:S18–21. . 8581090

[8] Poehlman ET, Melby CL, Goran MI. The impact of exercise and diet restriction on daily energy expenditure. Sports medicine. 1991;11(2):78–101. . 2017606 10.2165/00007256-199111020-00002

[9] Stanford KI, Middelbeek RJ, Goodyear LJ. Exercise Effects on White Adipose Tissue: Beiging and Metabolic Adaptations. Diabetes. 2015;64(7):2361–8. 10.2337/db15-0227 26050668 PMC4477356

[10] Stanford KI, Middelbeek RJ, Townsend KL, Lee MY, Takahashi H, So K, et al A novel role for subcutaneous adipose tissue in exercise-induced improvements in glucose homeostasis. Diabetes. 2015;64(6):2002–14. 10.2337/db14-0704 25605808 PMC4439563

[11] Rosen ED, Spiegelman BM. Molecular regulation of adipogenesis. Annual review of cell and developmental biology. 2000;16:145–71. 10.1146/annurev.cellbio.16.1.145 . 11031233

[12] Arner P, Spalding KL. Fat cell turnover in humans. Biochemical and biophysical research communications. 2010;396(1):101–4. Epub 2010/05/25. 10.1016/j.bbrc.2010.02.165 . 20494119

[13] Zeve D, Tang W, Graff J. Fighting fat with fat: the expanding field of adipose stem cells. Cell stem cell. 2009;5(5):472–81. Epub 2009/11/10. 10.1016/j.stem.2009.10.014 19896439 PMC2876189

[14] Tang W, Zeve D, Suh JM, Bosnakovski D, Kyba M, Hammer RE, et al White fat progenitor cells reside in the adipose vasculature. Science. 2008;322(5901):583–6. Epub 2008/09/20. 10.1126/science.1156232 18801968 PMC2597101

[15] Joe AW, Yi L, Even Y, Vogl AW, Rossi FM. Depot-specific differences in adipogenic progenitor abundance and proliferative response to high-fat diet. Stem cells. 2009;27(10):2563–70. Epub 2009/08/07. 10.1002/stem.190 . 19658193

[16] Sakurai T, Endo S, Hatano D, Ogasawara J, Kizaki T, Oh-ishi S, et al Effects of exercise training on adipogenesis of stromal-vascular fraction cells in rat epididymal white adipose tissue. Acta physiologica. 2010;200(4):325–38. Epub 2010/07/02. 10.1111/j.1748-1708.2010.02159.x . 20590530

[17] Henriksson J. Effects of physical training on the metabolism of skeletal muscle. Diabetes care. 1992;15(11):1701–11. . 1468304 10.2337/diacare.15.11.1701

[18] Bassel-Duby R, Olson EN. Signaling pathways in skeletal muscle remodeling. Annual review of biochemistry. 2006;75:19–37. Epub 2006/06/08. 10.1146/annurev.biochem.75.103004.142622 . 16756483

[19] Kriketos AD, Pan DA, Sutton JR, Hoh JF, Baur LA, Cooney GJ, et al Relationships between muscle membrane lipids, fiber type, and enzyme activities in sedentary and exercised rats. The American journal of physiology. 1995;269(5 Pt 2):R1154–62. . 7503305 10.1152/ajpregu.1995.269.5.R1154

[20] Rockl KS, Hirshman MF, Brandauer J, Fujii N, Witters LA, Goodyear LJ. Skeletal muscle adaptation to exercise training: AMP-activated protein kinase mediates muscle fiber type shift. Diabetes. 2007;56(8):2062–9. 10.2337/db07-0255 . 17513699

[21] Pedersen BK, Febbraio MA. Muscles, exercise and obesity: skeletal muscle as a secretory organ. Nature reviews Endocrinology. 2012;8(8):457–65. 10.1038/nrendo.2012.49 . 22473333

[22] Quiat D, Voelker KA, Pei J, Grishin NV, Grange RW, Bassel-Duby R, et al Concerted regulation of myofiber-specific gene expression and muscle performance by the transcriptional repressor Sox6. Proceedings of the National Academy of Sciences of the United States of America. 2011;108(25):10196–201. Epub 2011/06/03. 10.1073/pnas.1107413108 21633012 PMC3121857

[23] van Rooij E, Quiat D, Johnson BA, Sutherland LB, Qi X, Richardson JA, et al A family of microRNAs encoded by myosin genes governs myosin expression and muscle performance. Developmental cell. 2009;17(5):662–73. Epub 2009/11/20. 10.1016/j.devcel.2009.10.013 19922871 PMC2796371

[24] Seo J, Fortuno ES 3rd, Suh JM, Stenesen D, Tang W, Parks EJ, et al Atf4 regulates obesity, glucose homeostasis, and energy expenditure. Diabetes. 2009;58(11):2565–73. Epub 2009/08/20. 10.2337/db09-0335 19690063 PMC2768187

[25] Lapid K, Lim A, Clegg DJ, Zeve D, Graff JM. Oestrogen signalling in white adipose progenitor cells inhibits differentiation into brown adipose and smooth muscle cells. Nature communications. 2014;5:5196 10.1038/ncomms6196 . 25330806 PMC4770882

[26] Wu H, Rothermel B, Kanatous S, Rosenberg P, Naya FJ, Shelton JM, et al Activation of MEF2 by muscle activity is mediated through a calcineurin-dependent pathway. The EMBO journal. 2001;20(22):6414–23. Epub 2001/11/15. 10.1093/emboj/20.22.6414 11707412 PMC125719

[27] McKay RM, McKay JP, Avery L, Graff JM. C elegans: a model for exploring the genetics of fat storage. Developmental cell. 2003;4(1):131–42. Epub 2003/01/18. . 12530969 10.1016/s1534-5807(02)00411-2 PMC4445237

[28] Tang W, Zeve D, Seo J, Jo AY, Graff JM. Thiazolidinediones regulate adipose lineage dynamics. Cell metabolism. 2011;14(1):116–22. Epub 2011/07/05. 10.1016/j.cmet.2011.05.012 21723509 PMC3163675

[29] Zeve D, Seo J, Suh JM, Stenesen D, Tang W, Berglund ED, et al Wnt signaling activation in adipose progenitors promotes insulin-independent muscle glucose uptake. Cell metabolism. 2012;15(4):492–504. Epub 2012/04/10. 10.1016/j.cmet.2012.03.010 22482731 PMC3325026

[30] Rigamonti A, Brennand K, Lau F, Cowan CA. Rapid cellular turnover in adipose tissue. PloS one. 2011;6(3):e17637 Epub 2011/03/17. 10.1371/journal.pone.0017637 21407813 PMC3047582

[31] Spalding KL, Arner E, Westermark PO, Bernard S, Buchholz BA, Bergmann O, et al Dynamics of fat cell turnover in humans. Nature. 2008;453(7196):783–7. Epub 2008/05/06. 10.1038/nature06902 . 18454136

[32] Jo J, Gavrilova O, Pack S, Jou W, Mullen S, Sumner AE, et al Hypertrophy and/or Hyperplasia: Dynamics of Adipose Tissue Growth. PLoS computational biology. 2009;5(3):e1000324 10.1371/journal.pcbi.1000324 19325873 PMC2653640

[33] Kim SM, Lun M, Wang M, Senyo SE, Guillermier C, Patwari P, et al Loss of white adipose hyperplastic potential is associated with enhanced susceptibility to insulin resistance. Cell metabolism. 2014;20(6):1049–58. 10.1016/j.cmet.2014.10.010 . 25456741 PMC4715375

[34] Strissel KJ, Stancheva Z, Miyoshi H, Perfield JW 2nd, DeFuria J, Jick Z, et al Adipocyte death, adipose tissue remodeling, and obesity complications. Diabetes. 2007;56(12):2910–8. 10.2337/db07-0767 . 17848624

[35] Tchoukalova YD, Fitch M, Rogers PM, Covington JD, Henagan TM, Ye J, et al In vivo adipogenesis in rats measured by cell kinetics in adipocytes and plastic-adherent stroma-vascular cells in response to high-fat diet and thiazolidinedione. Diabetes. 2012;61(1):137–44. 10.2337/db10-1768 22124466 PMC3237665

[36] Wang QA, Tao C, Gupta RK, Scherer PE. Tracking adipogenesis during white adipose tissue development, expansion and regeneration. Nature medicine. 2013;19(10):1338–44. 10.1038/nm.3324 23995282 PMC4075943

[37] Jeffery E, Church CD, Holtrup B, Colman L, Rodeheffer MS. Rapid depot-specific activation of adipocyte precursor cells at the onset of obesity. Nature cell biology. 2015 10.1038/ncb3122 . 25730471 PMC4380653

[38] Wang YX, Zhang CL, Yu RT, Cho HK, Nelson MC, Bayuga-Ocampo CR, et al Regulation of muscle fiber type and running endurance by PPARdelta. PLoS biology. 2004;2(10):e294 Epub 2004/08/26. 10.1371/journal.pbio.0020294 15328533 PMC509410

[39] Narkar VA, Fan W, Downes M, Yu RT, Jonker JW, Alaynick WA, et al Exercise and PGC-1alpha-independent synchronization of type I muscle metabolism and vasculature by ERRgamma. Cell metabolism. 2011;13(3):283–93. Epub 2011/03/02. 10.1016/j.cmet.2011.01.019 21356518 PMC3084588

[40] Bostrom P, Wu J, Jedrychowski MP, Korde A, Ye L, Lo JC, et al A PGC1-alpha-dependent myokine that drives brown-fat-like development of white fat and thermogenesis. Nature. 2012;481(7382):463–8. 10.1038/nature10777 22237023 PMC3522098

[41] Kazanskaya O, Ohkawara B, Heroult M, Wu W, Maltry N, Augustin HG, et al The Wnt signaling regulator R-spondin 3 promotes angioblast and vascular development. Development. 2008;135(22):3655–64. 10.1242/dev.027284 . 18842812

[42] Ross SE, Hemati N, Longo KA, Bennett CN, Lucas PC, Erickson RL, et al Inhibition of adipogenesis by Wnt signaling. Science. 2000;289(5481):950–3. Epub 2000/08/11. . 10937998 10.1126/science.289.5481.950

[43] Bradley RL, Jeon JY, Liu FF, Maratos-Flier E. Voluntary exercise improves insulin sensitivity and adipose tissue inflammation in diet-induced obese mice. American journal of physiology Endocrinology and metabolism. 2008;295(3):E586–94. 10.1152/ajpendo.00309.2007 18577694 PMC2536732

[44] Carpenter KC, Strohacker K, Breslin WL, Lowder TW, Agha NH, McFarlin BK. Effects of exercise on weight loss and monocytes in obese mice. Comp Med. 2012;62(1):21–6. 22330647 PMC3276388

[45] Kawanishi N, Yano H, Yokogawa Y, Suzuki K. Exercise training inhibits inflammation in adipose tissue via both suppression of macrophage infiltration and acceleration of phenotypic switching from M1 to M2 macrophages in high-fat-diet-induced obese mice. Exerc Immunol Rev. 2010;16:105–18. . 20839495

[46] Sertie RA, Andreotti S, Proenca AR, Campana AB, Lima-Salgado TM, Batista ML Jr., et al Cessation of physical exercise changes metabolism and modifies the adipocyte cellularity of the periepididymal white adipose tissue in rats. J Appl Physiol (1985). 2013;115(3):394–402. 10.1152/japplphysiol.01272.2012 . 23703117

[47] Cassandra RP, Ghosh P, Tedeschi J, Gunasekara G, Broderick TL. Urinary corticosterone and normetanephrine levels after voluntary wheel and forced treadmill running in the db/db mouse. Journal of Diabetes Mellitus. 2011;1(4):71–8. 10.4236/jdm.2011.14011

[48] Sennott J, Morrissey J, Standley PR, Broderick TL. Treadmill exercise training fails to reverse defects in glucose, insulin and muscle GLUT4 content in the db/db mouse model of diabetes. Pathophysiology. 2008;15(3):173–9. 10.1016/j.pathophys.2008.06.001 . 18653321

[49] Terjung R. Endocrine response to exercise. Exerc Sport Sci Rev. 1979;7:153–80. . 399464

[50] Bunt JC. Hormonal alterations due to exercise. Sports medicine. 1986;3(5):331–45. . 3529282 10.2165/00007256-198603050-00003

[51] Popovic V, Duntas LH. Leptin TRH and ghrelin: influence on energy homeostasis at rest and during exercise. Horm Metab Res. 2005;37(9):533–7. 10.1055/s-2005-870418 . 16175489

