# Supplementary figures and images for: Exercise-Induced Skeletal Muscle Adaptations Alter the Activity of Adipose Progenitor Cells
Source: PLoS One. 2016 Mar 25;11(3):e0152129. doi: 10.1371/journal.pone.0152129 (PMC4807773; doi:10.1371/journal.pone.0152129)

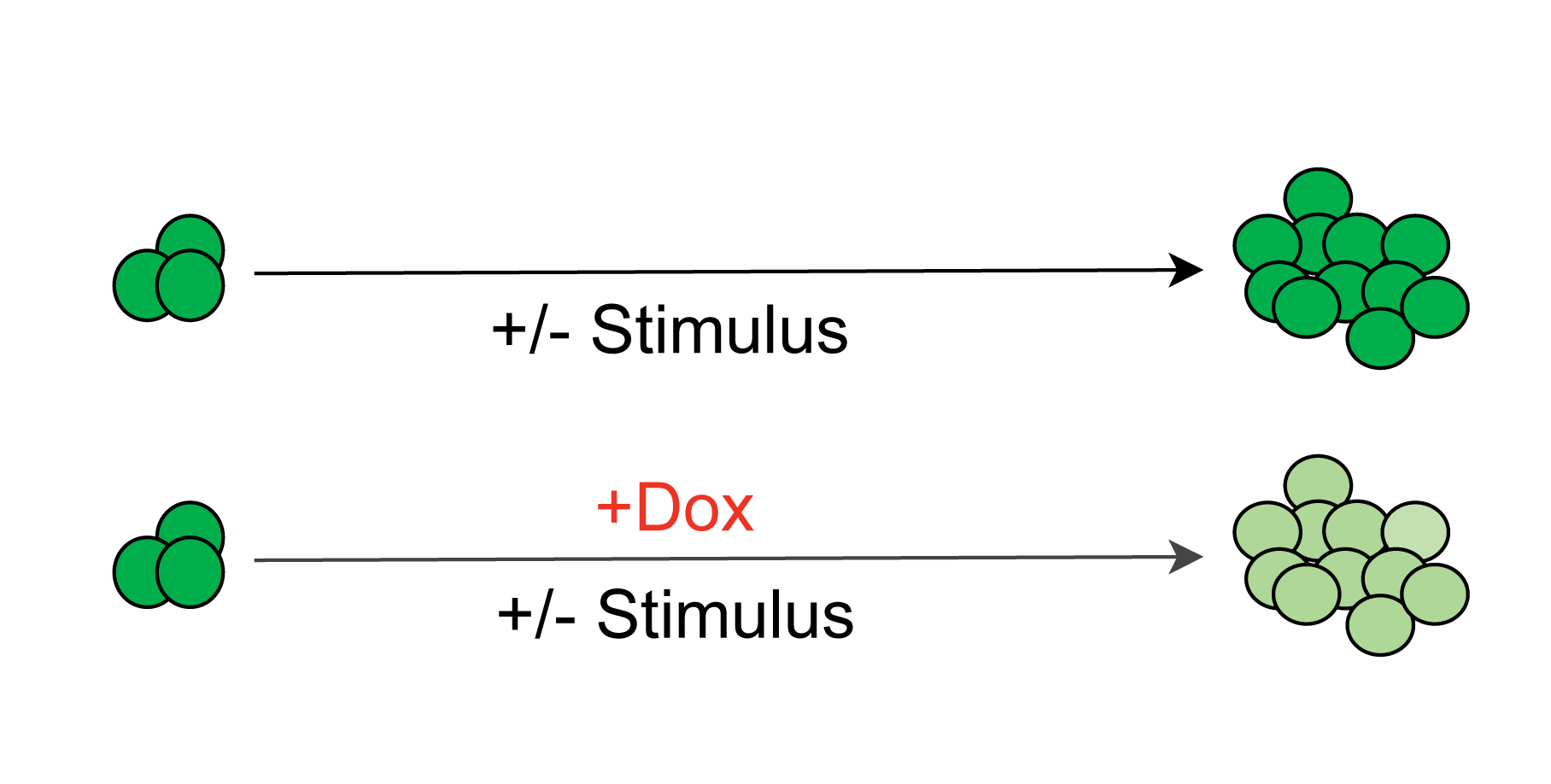

Supplement: S1 Fig — In the AdipoTrak mice, H2B-GFP expression is controlled by doxycycline (dox). In the absence of dox, H2B-GFP is similarly expressed in all adipose progenitor cells, regardless of stimulus (top row). Once dox is added to the system, H2B-GFP production is inhibited, with remaining H2B-GFP found within chromatin. H2B is a stable histone not broken down during mitosis. Therefore, division of adipose progenitors produces cells with decreased GFP intensity, as H2B-GFP is split between daughter cells. This allows us to investigate the proliferative capacity of the progenitor cells with or without stimulus through dox, since less GFP equates to increased proliferation (bottom row). (TIF) [file pone.0152129.s001.tif]

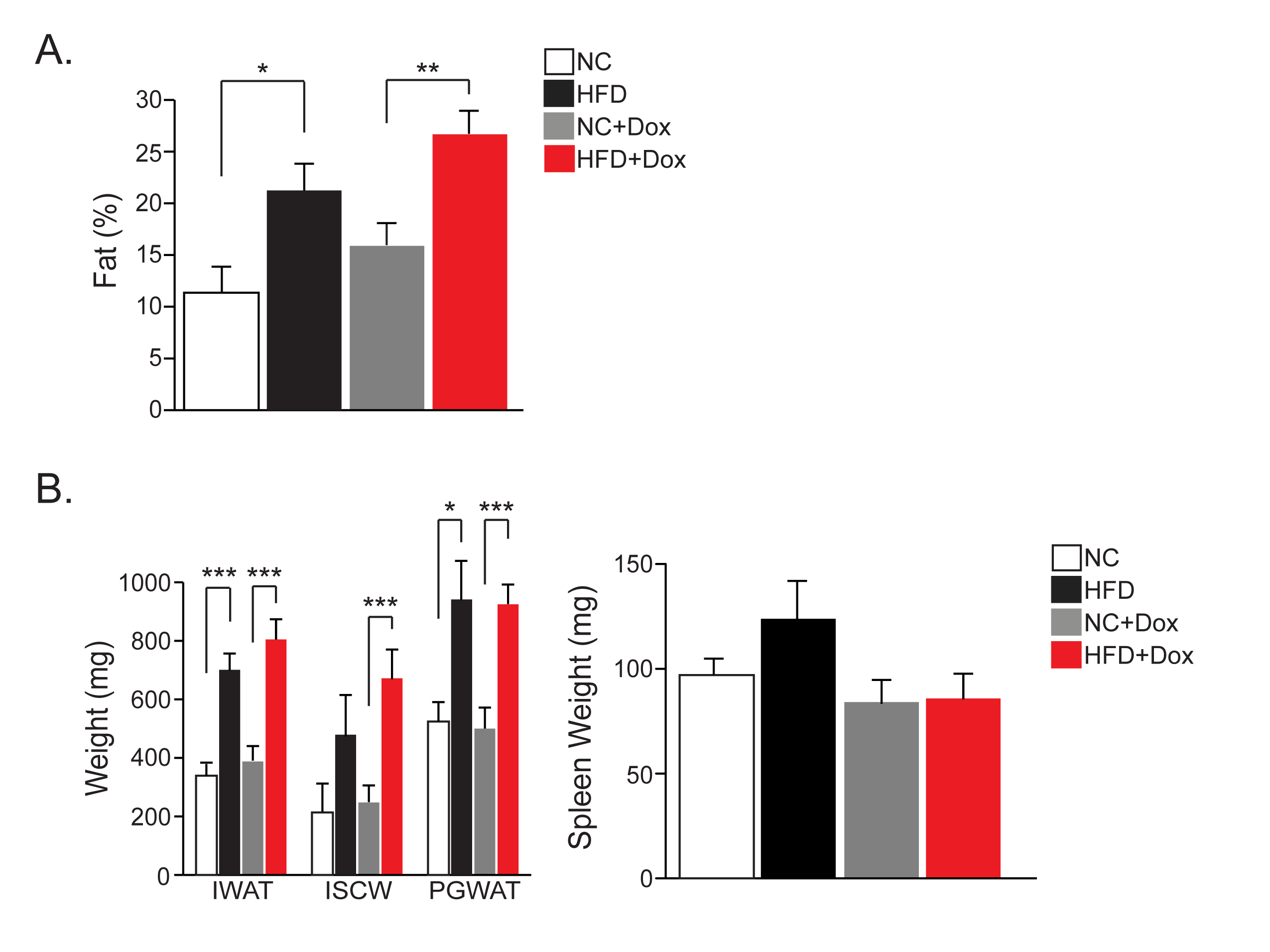

Supplement: S2 Fig — (A) Body fat percentage of mice on indicated treatment (NC: normal chow, HFD: high fat diet), either with dox (+Dox) or without dox. (B) (Left) Adipose depot weights of mice on indicated treatment (IWAT: inguinal white adipose tissue, ISCW: interscapular white adipose tissue, PGWAT: perigonadal white adipose tissue). (Right) Spleen weight of mice on indicated treatment. Representative studies on P120 males; n ≥ 16 per cohort, repeated ≥ 3 cohorts. Error bars indicate SEM. Statistical significance assessed by two-tailed Student’s t-test. *p<0.05, **p<0.01, ***p<0.001. (TIF) [file pone.0152129.s002.tif]

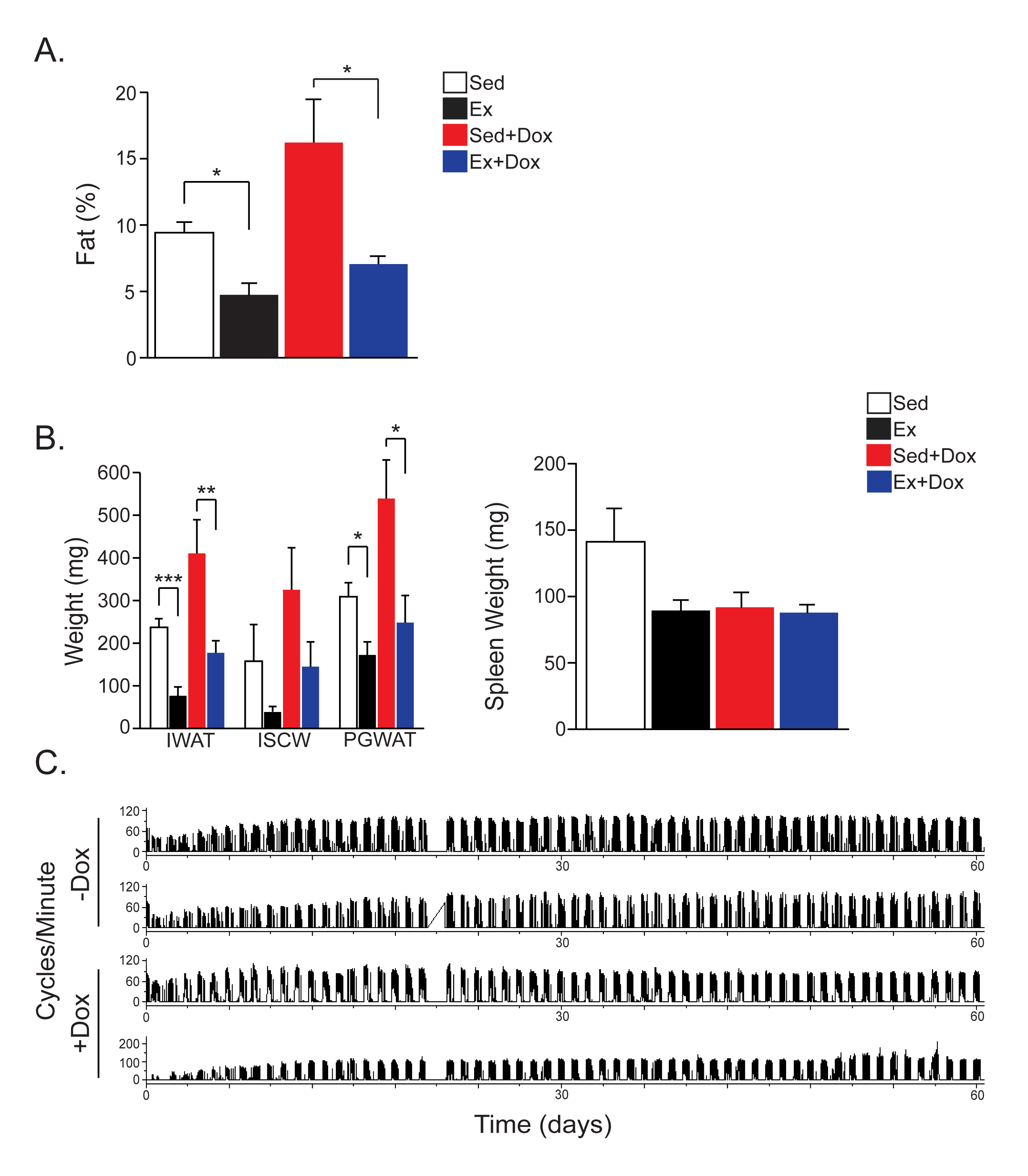

Supplement: S3 Fig — (A) Body fat percentage of mice on indicated treatment (Sed: sedentary, Ex: exercise), either with dox (+Dox) or without dox. (B) (Left) Adipose depot weights of mice on indicated treatment (IWAT: inguinal white adipose tissue, ISCW: interscapular white adipose tissue, PGWAT: perigonadal white adipose tissue). (Right) Spleen weight of mice on indicated treatment. (C) Rate of wheel revolutions (cycles/min) of indicated mice on running wheel. Representative studies on P120 males; n ≥ 16 per cohort, repeated ≥ 3 cohorts. Error bars indicate SEM. Statistical significance assessed by two-tailed Student’s t-test. *p<0.05, **p<0.01, ***p<0.001. (TIF) [file pone.0152129.s003.tif]

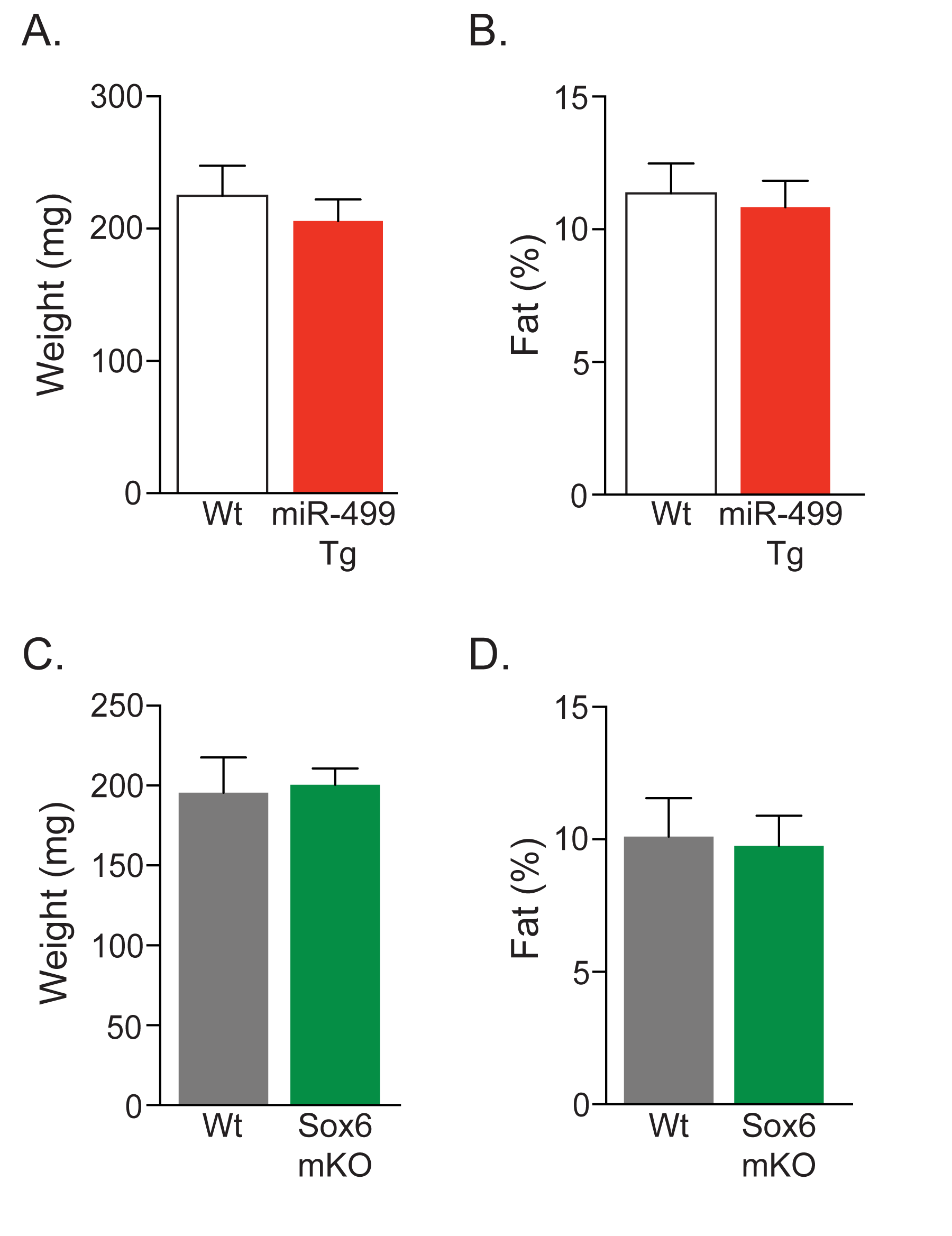

Supplement: S4 Fig — (A and B) Weight of inguinal fat depot (A) and total body fat content (B) of wild-type (Wt) and mir-499 Tg mice. (C and D) Weight of inguinal fat depot (C) and total body fat content (D) of Wt and Sox6 mKO mice. Representative studies on P60 females; n ≥ 8 per cohort, repeated ≥ 3 cohorts. Error bars indicate SEM. (TIF) [file pone.0152129.s004.tif]

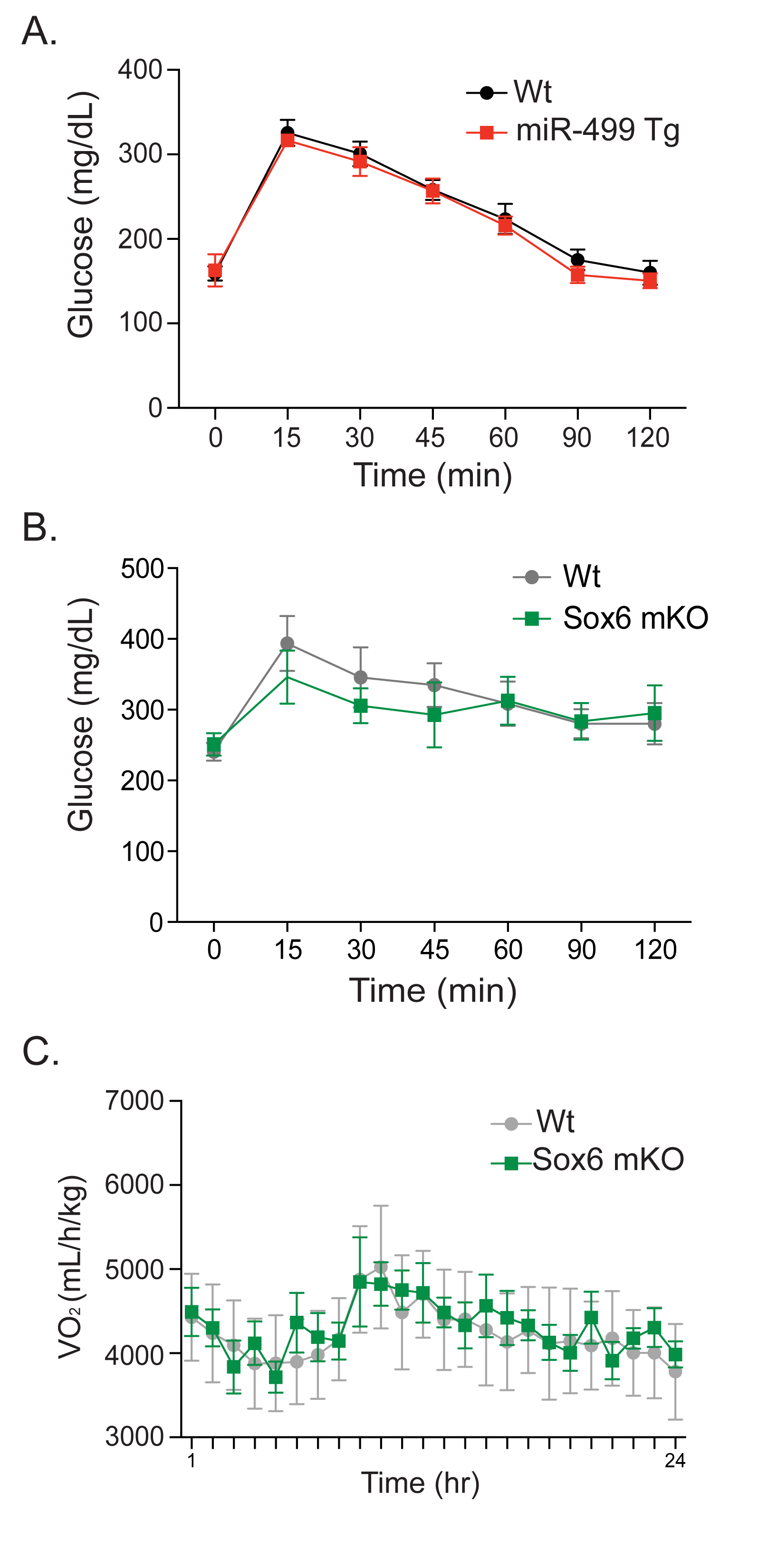

Supplement: S5 Fig — (A) Glucose tolerance test (GTT) of wild-type (Wt) and mir-499 Tg mice. (B) GTT of Wt and Sox6 mKO mice. (C) Oxygen consumption (VO2) of Wt and Sox6 mKO mice over a 24-hour period. Representative studies on P60 females; n ≥ 8 per cohort, repeated ≥ 3 cohorts. Error bars indicate SEM. Statistical significance assessed by two-tailed Student’s t-test. (TIF) [file pone.0152129.s005.tif]

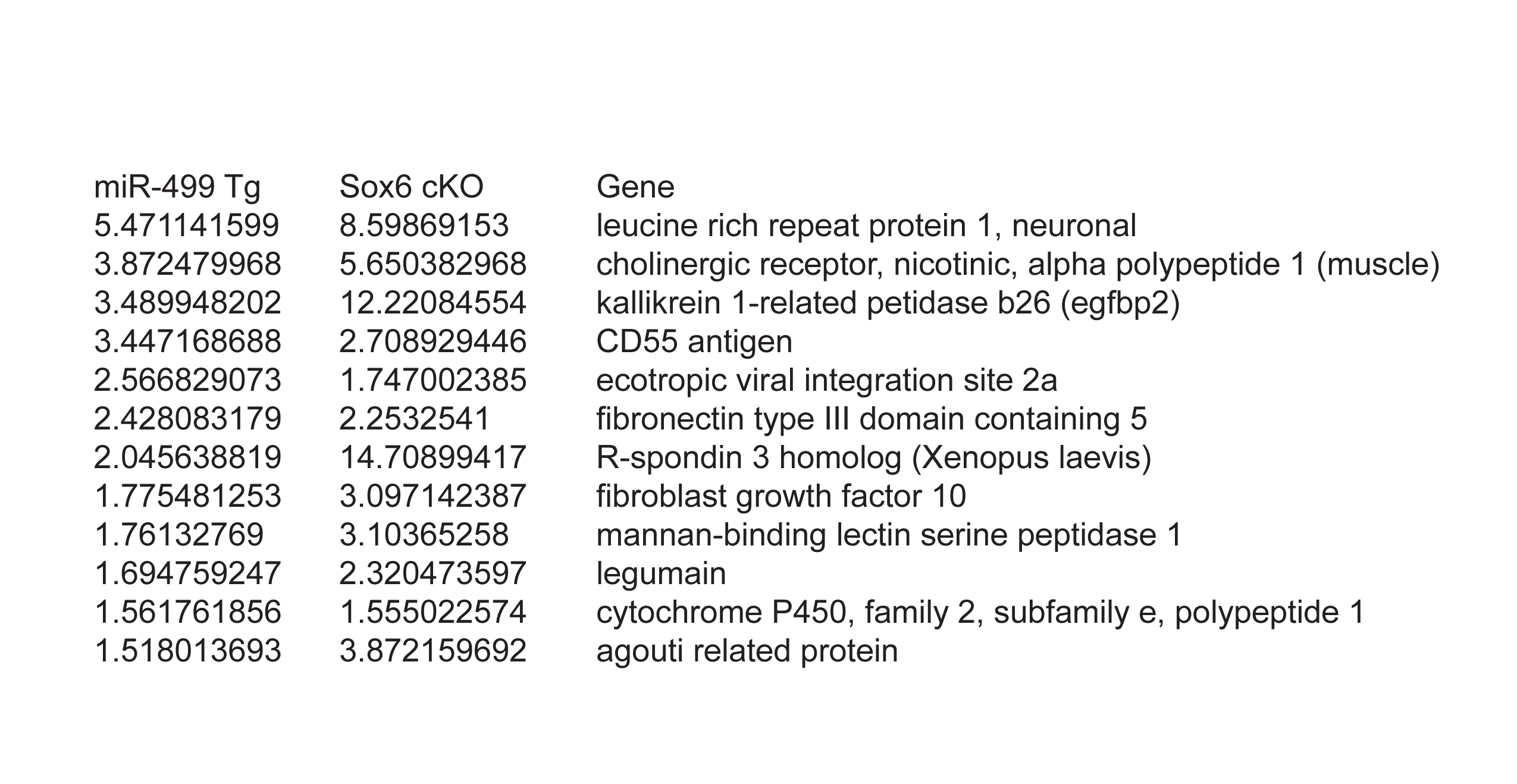

Supplement: S1 Table — Microarray analysis was performed on both mir-499 Tg and Sox6 mKO skeletal muscle compared to wild-type. Genes encoding a signal peptide with a greater than 2-fold expression in either mutant strain are shown. (TIF) [file pone.0152129.s006.tif]
